# Naturally Acquired Immune Responses to *P. vivax* Merozoite Surface Protein 3α and Merozoite Surface Protein 9 Are Associated with Reduced Risk of *P. vivax* Malaria in Young Papua New Guinean Children

**DOI:** 10.1371/journal.pntd.0002498

**Published:** 2013-11-14

**Authors:** Danielle I. Stanisic, Sarah Javati, Benson Kiniboro, Enmoore Lin, Jianlin Jiang, Balwan Singh, Esmeralda V. S. Meyer, Peter Siba, Cristian Koepfli, Ingrid Felger, Mary R. Galinski, Ivo Mueller

**Affiliations:** 1 Walter and Eliza Hall Institute, Parkville, Australia; 2 Papua New Guinea Institute of Medical Research, Madang, Papua New Guinea; 3 Emory Vaccine Center, Yerkes National Primate Research Centre, Emory University, Atlanta, Georgia, United States of America; 4 Swiss Tropical Institute and Public Health Institute, Basel, Switzerland; 5 University of Basel, Basel, Switzerland; 6 Department of Medicine, Division of Infectious Disease, Emory University, Atlanta, Georgia, United States of America; 7 Barcelona Centre for International Health Research (CRESIB, Hospital Clínic-Universitat de Barcelona), Barcelona, Spain; Queensland Institute for Medical Research, Australia

## Abstract

**Background:**

*Plasmodium vivax* is the most geographically widespread human malaria parasite. Cohort studies in Papua New Guinea have identified a rapid onset of immunity against vivax-malaria in children living in highly endemic areas. Although numerous *P. vivax* merozoite antigens are targets of naturally acquired antibodies, the role of many of these antibodies in protective immunity is yet unknown.

**Methodology/Principal Findings:**

In a cohort of children aged 1–3 years, antibodies to different regions of Merozoite Surface Protein 3α (PvMSP3α) and Merozoite Surface Protein 9 (PvMSP9) were measured and related to prospective risk of *P. vivax* malaria during 16 months of active follow-up. Overall, there was a low prevalence of antibodies to PvMSP3α and PvMSP9 proteins (9–65%). Antibodies to the PvMSP3α N-terminal, Block I and Block II regions increased significantly with age while antibodies to the PvMSP3α Block I and PvMSP9 N-terminal regions were positively associated with concurrent *P. vivax* infection. Independent of exposure (defined as the number of genetically distinct blood-stage infection acquired over time (_mol_FOB)) and age, antibodies specific to both PvMSP3α Block II (adjusted incidence ratio (aIRR) = 0.59, p = 0.011) and PvMSP9 N-terminus (aIRR = 0.68, p = 0.035) were associated with protection against clinical *P. vivax* malaria. This protection was most pronounced against high-density infections. For PvMSP3α Block II, the effect was stronger with higher levels of antibodies.

**Conclusions:**

These results indicate that PvMSP3α Block II and PvMSP9 N-terminus should be further investigated for their potential as *P. vivax* vaccine antigens. Controlling for _mol_FOB assures that the observed associations are not confounded by individual differences in exposure.

## Author Summary

*Plasmodium vivax* is the most geographically widespread human malaria parasite. In highly endemic areas such as Papua New Guinea, a very rapid onset of immunity against vivax-malaria is observed. Although it is known that numerous *P. vivax* merozoite antigens are targets of naturally acquired antibodies, the role of many of these antibodies in protective immunity is yet unknown. In a cohort of 183 children aged 1–3 years, we now show that the presence of antibodies to Merozoite Surface Protein 3α (PvMSP3α) and Merozoite Surface Protein 9 (PvMSP9) are associated with a significant reduction in the burden *P. vivax* malaria. Antibodies increased with age and in the presence of concurrent *P. vivax* infections. After adjusting for both age and individual differences in exposure, the strongest reductions in risk were seen in children with antibodies to PvMSP3α Block II (41% reduction, p = 0.001) and PvMSP9 N-terminal region. (32% reduction, p = 0.035). These results indicate that PvMSP3α Block II and PvMSP9 N-terminus should be further investigated for their potential as *P. vivax* vaccine antigens.

## Introduction

Historically, most malaria vaccine research and development has been focused on *Plasmodium falciparum*. However, the importance of developing a *P. vivax* specific or combination *P. falciparum*/*P. vivax* vaccine is increasingly being recognised [1]. *P. vivax* is the most geographically widespread malaria parasite with up to 2.5 billion people at risk and an estimated 80–300 million clinical cases every year [2]. It is not the benign parasite it was long assumed to be; while severe manifestations are less common [3], there is a spectrum of severe disease associated with *P. vivax* infection that in many ways resembles that seen with *P. falciparum* [4]. Furthermore, case fatality rates associated with severe *P. vivax* or mixed *P. falciparum*/*P. vivax* infections are comparable with *P. falciparum* [3], [5], [6]. Unique aspects of the biology of this particular species of *Plasmodium* make it a challenge to treat and eradicate with currently available strategies [7], [8], [9], [10]. *P. vivax* forms dormant stages in the liver (hypnozoites), which can result in relapses following effective anti-malarial treatment of blood-stage infection [8]. It is also able to produce gametocytes early in infection which may appear in the peripheral circulation before the development of clinical symptoms [9]. Therefore, an infected, asymptomatic but untreated individual serves as a ‘reservoir’, maintaining successful transmission of the parasite. An effective *P. vivax* vaccine is a desirable, additional tool for *P. vivax* elimination.

Prioritisation of malaria vaccine candidates is informed by their site and stage expression, apparent function *in vitro* and role in protective immunity in malaria exposed populations. The identification and subsequent development of candidates for a *P. vivax* specific vaccine has been challenging due to a number of practical factors including the lack of a reliable *in vitro* culture system and limited data with respect to antigen diversity. Several antigens expressed during the blood stage of *P. vivax* infection have been identified as potential vaccine candidates including the Duffy Binding Protein (PvDBP, one of the primary erythrocyte invasion ligands), Merozoite Surface Protein 3 (PvMSP3) and Merozoite Protein 9 (PvMSP9) [11]–[14]. Antibodies against the most studied *P. vivax* vaccine candidate, the PvDBP, have been shown to inhibit binding of the parasite to receptors on the red blood cell and have been associated with protection [15]. PvDBP Region II (RII), the critical region for binding, is however quite polymorphic and the protection observed in this study had a degree of strain specificity [15]. This suggests that a vaccine based on PvDBP RII should either target conserved epitopes and be able to induce broadly inhibitory antibodies as recently demonstrated *in vitro* [16] or it may need to include multiple allelic types. Additionally, recent observations that *P. vivax* can utilise a Duffy antigen-independent invasion pathway and invade Duffy-negative red cells [17] suggests that a vaccine based solely on the PvDBP will not be effective against all *P. vivax* strains. Consequently, it is essential that additional *P. vivax* antigens are also critically assessed for their potential as vaccine candidates.

The *P. vivax* MSP3 multigene family is expressed during the erythrocytic stage of the life-cycle, with the majority of the proteins expressed in the schizont stage when merozoites are being formed [13], [14], [18]. Members are structurally related to *P. falciparum* MSP3, which has been shown to mediate antibody-dependent cellular-mediated inhibition [19] and is a vaccine candidate that showed some efficacy in human trials following preliminary analyses [20]. PvMSP3 lacks a hydrophobic region which indicates the presence of a transmembrane domain that could link it to the merozoite surface, rather it is thought to associate with other surface anchored proteins [13]. Its central alanine-rich domain with heptad repeats is predicted to form coiled-coil tertiary structures which mediate protein-protein interactions.

One of the members of the PvMSP3 family, originally identified as PvMSP3α, has been the focus of several specific bodies of research. Expression of PvMSP3α has been detected in trophozoites and schizonts and is displayed at the surface of merozoites [18], [21]. This protein is highly polymorphic and it has therefore been used as a molecular marker in *P. vivax* epidemiological and population studies [22], [23], [24], [25]. However, the hydrophilic, extreme N-terminal and the acidic C-terminal domains of the protein are relatively conserved [26]. Polymorphisms are clustered in specific domains, mainly confined to the N-terminal half of the central alanine-rich coiled-coil domain (designated as Block I, residues 104–396) while the C-terminal portion of this domain (designated Block II, residues 434–687) displays less variability [26]. Block I may be deleted in some isolates while retention of the relatively highly conserved Block II appears to be necessary. Due to the relative conservation of Block II of the alanine rich domain and the acidic C-terminal region, across a range of geographically distinct isolates, it has been suggested that vaccine-based research should focus on these regions [26]. PvMSP3α specific antibodies have been detected in naturally exposed individuals resident in a malaria endemic area of the Brazilian Amazon [21], and a number of linear B cell epitopes defined, located primarily in the 2 blocks of repeats [27].

PvMSP9 is also expressed during schizogony and is associated with the surface of the merozoite [29], [30]. The deduced protein contains a hydrophobic signal sequence, highly conserved N-terminal domain with a cluster of 4 cysteines and a C-terminal region containing 2 species-specific blocks of repeated amino acids, designated PvMSP9-RI and PvMSP9-RII [29]. Its importance as a vaccine candidate has been highlighted by the ability of a PvMSP9 monoclonal antibody to block the entry of *P. vivax* into erythrocytes [30]. Studies examining the immunogenicity of different regions of PvMSP9 have demonstrated the presence of both antibody and T cell responses specific for this protein in individuals resident in malaria endemic areas [12], [31], [32].

Recent cohort studies in Papua New Guinea (PNG), where both *P. falciparum* and *P. vivax* co-exist, have identified an age-dependent onset of immunity to the different *Plasmodium* species. The incidence of *P. vivax* attributable illness peaks in the second year of life, compared to *P. falciparum* where it continues to increase until the 4^th^ year of life, suggesting that immunity against *P. vivax* appears to be acquired at a younger age than that seen with *P. falciparum* [33]. This immunity is associated with an increased ability to control parasite densities so that they remain below the threshold above which symptoms are apparent. Despite these observations, little is known about immune responses against *P. vivax* antigens in young children and how these may contribute to the acquisition of protective immunity. To address this, we examined associations between antibodies to different regions of the blood-stage antigens PvMSP3α and PvMSP9 and prospective risk of *P. vivax* malaria in a cohort of children aged 1–3 years residing in a malaria endemic region of PNG.

## Materials and Methods

### Study description

This study was conducted in a rural area near Maprik, East Sepik Province, Papua New Guinea. A detailed description of the study is given elsewhere [33]. Briefly, 264 study participants aged 1–3 years (median 1.70; range 0.9–3.1 years) were enrolled between March and September 2006 and venous blood collected. Of these, 190 were enrolled at the study start and 74 over the subsequent 6 months. Antibody assays were performed using samples from 183 of the 190 children enrolled at the study start; all data presented for the current analysis relates to these 183 children only. Following enrolment, children were clinically examined every 2 weeks for signs and symptoms of malaria for a period of up to 16 months (until July 2007). In addition, children were actively checked every 8 weeks, with visits scheduled over 2 consecutive days (with 2 samples collected 24 hours apart) to improve detection of low-level infection. A passive case detection system was maintained at the local health centres and aid post throughout the entire study period. At each episode of febrile illness, a blood sample was collected, a rapid diagnostic test (RDT) was performed and haemoglobin measured using Hemacue (Angholm, Sweden). Anti-malarial treatment with Coartem® (Novartis, Switzerland) was administered to any individual with a positive RDT or if haemoglobin levels were <7.5 g/dl. In children with a negative RDT, blood slides were read within 24 hours and microscopy positive children were treated with Coartem.

For the current analysis, a symptomatic episode of *P. vivax* malaria was defined as the presence of fever plus parasitemia >500 parasites/µl [34]. Parasitemia (ie absence/presence of parasite) was determined by a semi-quantitative post-PCR ligase detection reaction-fluorescent microsphere assay (LDR-FMA) [35] and light microscopy was used to determine parasite density. All analyses were performed using parasitemia determined by LDR-FMA unless otherwise indicated.

Written informed consent was obtained from all parents or guardians prior to recruitment of each child. Scientific approval and ethical clearance for the study was obtained from the Medical Research and Advisory Committee (MRAC) of the Ministry of Health in PNG and the Human Research Ethics Committee, the Walter and Eliza Hall Institute.

### Antigens

PvMSP3α recombinant proteins, representing the N-terminal (nucleotides 73–309), Block I (nucleotides 316–1242), Block II (nucleotides 1246–2058) and the C-terminal (nucleotides 2059–2353) regions were used. They were initially amplified from *P. vivax* (Belem strain), expressed as His-tag recombinant proteins and purified as previously described [27].

PvMSP9 recombinant proteins, representing the N-terminal region (aa 34–193) and the C-terminal region containing the repeated Blocks I and II (aa 729–972), were used. They were initially amplified from *P. vivax* (Belem strain), expressed as GST fusion proteins and purified as previously described [12], [31].

The proteins were assessed on SDS-PAGE gels and via western immunoblots using standard conditions. A single batch of each protein was used for this was well as earlier Brazilian studies [12], [31].

### Antibody assays

Samples collected from the enrolment bleed (n = 183) were used in an enzyme linked immunosorbent assay (ELISA). All available samples were tested for IgG. ELISAs were performed using established methods [36]. Ninety-six well plates (Nunc, Roskilde, Denmark) were coated with MSP3α and MSP9 recombinant proteins in PBS and incubated overnight at 4°C. For MSP9 proteins, GST alone was used as a control antigen. Skim milk-PBS-0.05% Tween was used for blocking and for diluting plasma and antibodies. Plasma was added in duplicate at previously determined dilutions. For measurement of total IgG, horseradish peroxidase-conjugated sheep anti-human IgG (Chemicon, Melbourne, Australia) was used at a dilution of 1, 2∶500. Finally, o-phenylenediamine dihydrochloride substrate (Sigma, Castle Hill, Australia) was added and the reaction stopped using 3M HCl with optical density determined at 492 nm. All samples were tested in duplicate. Standardization of the plates was achieved using positive control plasma pools on each plate. Background (determined from the wells with no plasma) was deducted from the mean of each sample and a cut-off threshold for positivity determined as the mean plus 3 standard deviations of negative control plasma samples (Australian residents) included in each assay. For MSP9 proteins, final OD values were determined by subtracting the mean OD value to GST alone from the mean OD value of the same plasma for the recombinant proteins.

### Measuring Force of Blood-stage infection (_mol_FOB)

The _mol_FOB was used to define the number of new *P. vivax* blood-stage clones acquired during the study follow-up period [37]. For genotyping individual *P. vivax* clones, the highly polymorphic molecular markers Merozoite Surface Protein 1 F3 fragment and the microsatellite MS16 were typed using capillary electrophoresis for precise fragment sizing. Details of the genotyping techniques have been described elsewhere [38].

### Statistical analyses

Antibody levels were not normally distributed, so non-parametric tests (Mann-Whitney U tests) were used for analyses of antibody titres. Differences in the prevalence of antibodies with age and infection status as well as associations between antibodies to different proteins were assessed using Chi-square test with the strength of the association between antibodies of different specificities measured by the phi coefficient (r_φ_). The association of antibody prevalence and parasite density were assessed using generalised estimating equation (GEE) models.

Children were followed up for a maximum of 8 periods during the study, each spanning 8–9 weeks and consisting of 3 fortnightly surveillance visits and each concluding with the collection of 2 blood samples 24 hours apart for active detection of malaria infection. Incidence of clinical malaria in each 8–9 week follow-up interval was estimated as previously described, with *P. vivax* clinical episodes defined as febrile illness (axillary temperature ≥37.5°C or history of fever in preceding 48 hrs) with a concurrent *P. vivax* parasitemia >500 parasites/µl. A negative binomial GEE model (based on XTNBREG procedure in STATA 12.0), an exchangeable correlation structure and semi-robust variance estimator were used for the analysis of incidence of *P. vivax* malaria. For each follow-up interval, children were considered at risk from the first day after the second or only blood sample for active follow-up was taken. Therefore, cross-sectional bleeds were considered as part of the preceding 8–9 week interval and clinical episodes detected during those cross-sectional bleeds (2 samples taken 24 hours apart) were included in that interval. Children were not considered at risk for 2 weeks after treatment with Coartem®.

Three different models were used to assess the association of antibodies with protection: i) ‘crude’: adjustment only for seasonal (month, year) and spatial variation (village or residence) as well as for individual differences in exposure: aIRR_(exp)_; ii) age-adjusted: additional adjustment for age of child (as a correlate of overall immune status): aIRR_(exp+age)_, iii) multivariate age-adjusted: multivariate analyses of all antibodies univariately associated with protection: aIRR(_multi_), with the best model determined by backward elimination using Wald's Chi-square tests for individual variables.

Individual differences in exposure were described by the number of genetically distinct *P. vivax* clones a child acquired during 2 month intervals, expressed as the number of new blood-stage infections per unit of time. Samples from scheduled bleeds as well as morbidity surveillance were used. The force of infection for each child was therefore defined as the number of new blood-stage clones acquired per year at risk (i.e. the molecular force of infection _mol_FOB [39]). In order to improve the fit, _mol_FOB was cube-root transformed [37].

## Results

### *P. vivax* prevalence in the cohort

A total of 183 children 0.9–3.1 years (47.5% ≥21 months, 56.8% male) were enrolled in late March 2006 and actively followed for 16 months for the development of malaria infection. At enrolment, the prevalence of *P. vivax* was 39.9% and 49.2% by light microscopy (LM) and post-PCR LDR-FMA, respectively. During follow-up, *P. vivax* prevalence ranged from 59.9–79.2% by post-PCR LDR-FMA and 47.3–59.9% by LM. Children experienced an average of 2.47 (CI_95_ [2.15, 2.85]) *P. vivax* episodes with any level of parasitemia and 1.49 (CI_95_ [1.24, 1.79]) episodes with *P. vivax* >500 parasites/µl per year at risk. The patterns of *P. vivax* infection and disease in the immunology sub-cohort are therefore comparable to those observed in the entire cohort [33], [38].

### Presence of IgG antibodies and their association with age and infection status

**Figure 1:**
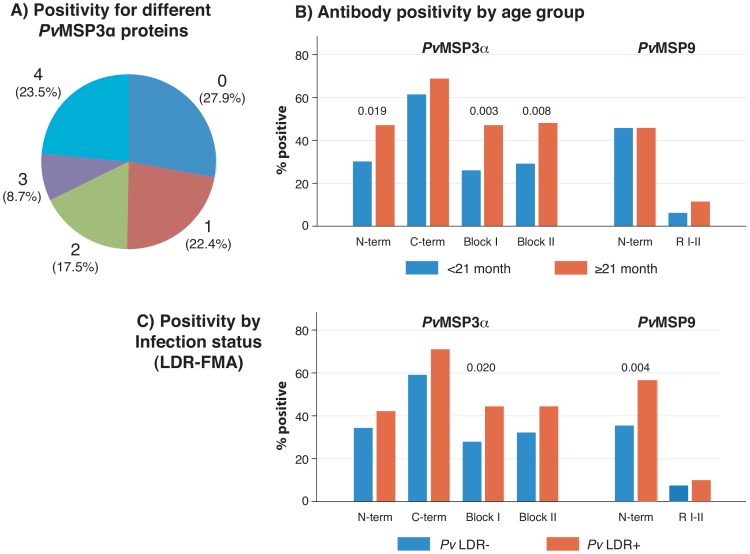
IgG positivity to different PvMSP3α and PvMSP9 proteins. (a) Cumulative IgG positivity for different PvMSP3α proteins. Data are plotted as the percentage of 183 individuals who are antibody positive for 0–4 of the proteins tested. (b) Associations between age and IgG positivity to PvMSP3α and PvMSP9 proteins. Children were divided into two age groups (<21 mths: n = 96, ≥21 mths: n = 87) to examine associations with age. P values≤0.05 were considered significant and are shown. (c) Associations between *P. vivax* infection status (post-PCR LDR-FMA positive: n = 90, negative: n = 93) and IgG positivity to PvMSP3α and PvMSP9 proteins. As indicated, the presence of *P. vivax* was determined by a semi-quantitative post-PCR ligase detection reaction-fluorescent microsphere assay (LDR-FMA). P values≤0.05 were considered significant and are shown.

The frequency of IgG responses at enrolment to the different antigens ranged from 8.7%–65% (Table 1). When comparing different regions of PvMSP3α, significantly more children had IgG antibodies to the C-terminal than to the N-terminal, Block I and Block II proteins of PvMSP3α (65.0% vs. 36.1–38.3%, p<0.001). The presence of antibodies to the different protein constructs derived from PvMSP3α were highly associated with each other (p<0.0001 for any pair) with the strongest association found between antibodies to Block II and Block I (r_φ_ = 0.67). Overall, 43 children (23.5%) had antibodies to all 4 PvMSP3α proteins while 51 (27.9%) had antibodies to none (Figure 1a).

**Table 1:** Optical density values (OD) as a measurement of total IgG to different PvMSP3α and PvMSP9 proteins.

	PvMSP3α				PvMSP9	
	N-terminal	C-terminal	Block I	Block II	N-terminal	RI-RII
**Median OD (IQR)**	0.26 [0.07,0.67]	0.33 [0.15,0.62]	0.28 [0.12,0.74]	0.27 [0.09,0.67]	0.39 [0.18,0.98]	0.05 [0.02,0.14]
**OD value ranges minimum-maximum**	0.01–2.25	0.03–1.63	0.01–1.74	0.02–1.60	0.03–2.35	0.00–2.00
**OD positivity cut-off** a	0.43	0.21	0.43	0.44	0.48	0.43
**Frequency of responders** b **(n)**	38.3% (70)	65.0% (119)	36.1% (66)	38.3% (70)	45.9% (84)	8.7% (16)

a The cut-off for positivity was determined as the mean+3 standard deviations of negative control plasma samples (Australian residents) included in each assay.

b Responders defined as individuals whose plasma OD value was above the cut-off for positivity for a given antigen.

Children ≥21 months were significantly more likely to have antibodies to the PvMSP3α N-terminal protein (Odds ratio (OR = 2.06, CI_95_ [1.08, 3.95], p = 0.019), Block I (OR = 2.53, CI_95_ [1.30, 4.95], p = 0.003) and Block II (OR = 2.27, CI_95_ [1.18, 4.37], p = 0.008) but not to the C-terminal protein (Figure 1b). Antibodies to Block I (OR = 2.06, CI_95_ [1.07, 4.00], p = 0.020) were also significantly more common in children with concurrent *P. vivax* infection (Figure 1c). There were no significant associations with antibody levels and either age or infection status among children that were antibody-positive for any of the PvMSP3α proteins (p>0.26).

Eighty-four (45.9%) children had antibodies to the PvMSP9 N-Terminal region, and 16 (8.7%) had antibodies to the MSP9 protein spanning RI-RII (Table 1). Children that were positive for any of the PvMSP3α proteins were more likely to also be positive for the PvMSP9 N-Terminus (OR = 2.30–4.78, p<0.009). Antibodies specific for the PvMSP9 N-Terminus were more common in children with concurrent *P. vivax* infection (OR = 2.38, CI_95_ [1.26, 4.51], p = 0.004). No other significant associations between antibodies specific for PvMSP9 derived proteins and either age or infection status were observed.

### Association between presence of antibodies and prospective risk of *P. vivax* malaria

When assessing associations between the presence of IgG antibodies specific for the different PvMSP3α and PvMSP9 proteins and prospective risk of *P. vivax* clinical episodes (>500 parasites/µl, Table 2) during the 16 months of follow-up, adjustments were made first for different measures of malaria exposure as described in the materials and methods section, designated aIRR(_exp_). This was followed by a further adjustment for age, designated aIRR(_exp+age_).

**Table 2:** Association between antibody positivity and protection against subsequent *P. vivax* malaria (density>500/µl).

		Models used to assess the association of antibodies with protection								
		‘Crude’^a^			Age-adjusted^b^			Multivariate^c^		
		aIRR_(exp)_	CI_95_	p -value	aIRR_(exp+age)_	CI_95_	p -value	aIRR_(multi)_	CI_95_	p -value
**PvMSP3α**	**N-term**	0.67	[0.44,1.00]	0.048	0.79	[0.54,1.15]	0.222			
	**C-term**	0.77	[0.54,1.12]	0.172	0.82	[0.58,1.16]	0.267			
	**Block I**	0.72	[0.50,1.04]	0.077	0.75	[0.53,1.07]	0.116			
	**Block II**	0.46	[0.31,0.68]	<0.001	0.53	[0.36,0.77]	0.001	0.59	[0.40,0.89]	0.011
**PvMSP9**	**N-term**	0.58	[0.40,0.84]	0.004	0.60	[0.42,0.85]	0.004	0.68	[0.47,0.97]	0.035
	**RI-RII**	0.81	[0.42,1.54]	0.515	0.97	[0.51,1.82]	0.915			

a Adjustment for season (month, year), spatial variation (village or residence) and individual differences in exposure (as measured by molecular force of blood-stage infection (_mol_FOB).

b Adjustment for age of child, season (month, year), spatial variation (village or residence) and individual differences in exposure (as measured by molecular force of blood-stage infection (_mol_FOB).

c Adjustment as performed for age-adjusted b. Multivariate analyses of antibodies univariately associated with protection.

After adjusting for malaria exposure, a significant decrease in the risk of clinical *P. vivax* malaria was associated with the presence of antibodies for PvMSP3α Block II (aIRR_(exp)_ = 0.46, p<0.001), PvMSP3α N-terminus (aIRR(_exp_) = 0.67, p = 0.048), and PvMSP9 N-Terminus (aIRR(_exp_) = 0.58, p = 0.004) (Table 2).

**Figure 2:**
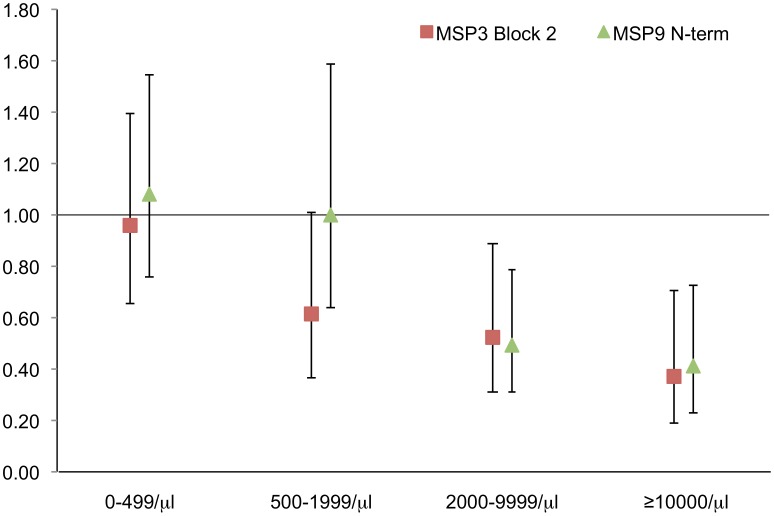
Association between antibodies to PvMSP3α(Block II) and PvMSP9(N-terminal) proteins and risk of *P. vivax* malaria. Data are plotted as exposure and age adjusted incidence rate ratios (aIRR(exp)) ± 95% confidence intervals for febrile episodes with different levels of concurrent *P. vivax* parasitaemia.

Following a further adjustment for age, a significantly decreased risk remained for individuals who were antibody positive for PvMSP3α Block II (aIRR_(exp+age)_ = 0.53, p = 0.001) and PvMSP9 N-Terminus (aIRR(_exp+age_) = 0.60, p = 0.004) (Table 2). For antibodies specific for both these proteins there was a tendency for protection to increase with increasing levels of parasitaemia (Figure 2). However, whereas the presence of antibodies for PvMSP3α Block II was associated with protection against all clinical episodes with a *P. vivax* parasitaemia ≥500/µl (p = 0.003–0.057), antibodies against PvMSP9 N-Terminus were associated only with significant protection against episodes with ≥2000/µl (p = 0.001–0.002) but not those with densities ranging from 500–1999/µl (p = 0.92).

To assess the effect of antibody levels on protection against risk of *P. vivax* clinical episodes, children who were antibody positive for PvMSP3α Block II or PvMSP9 N-Terminus were stratified into 2 equal sized groups: those with high levels and those with low levels of antibodies. High levels (OD>0.77) of PvMSP3α Block II specific antibodies were significantly associated with protection (aIRR = 0.42, p = 0.001) whereas, this was not observed with lower levels (IRR = 0.68, p = 0.114). No such differences were observed with antibodies to PvMSP9 N-Terminus.

In multivariate analyses, both antibodies to PvMSP3α Block II (aIRR(_multi_) = 0.59, p = 0.011) and PvMSP9 N-Terminus (aIRR(_multi_) = 0.68, p = 0.035) remained significantly associated with a reduced risk of *P. vivax* (Table 2). A significant interaction between the presence of antibodies to PvMSP9 N-Terminus and concurrent *P. vivax* infection was observed (X^2^ = 4.55, p = 0.033) with PvMSP9 N-Terminus antibodies associated with protection only in children without concurrent *P. vivax* infection (aIRR(_multi_) = 0.48, CI_95_[0.28, 0.80], p = 0.006), while in children with concurrent infection PvMSP9 N-Terminus, antibodies did not add any extra protection (aIRR(_multi_) = 1.03, CI_95_[0.62, 1.71], p = 0.9) besides that associated with concurrent infection itself (aIRR(_multi_) = 0.59, CI_95_[0.41, 0.87], p = 0.007).

As multivariate analyses examining the relationship between the presence of antibodies and risk of clinical *P.vivax* malaria showed a protective effect with only PvMSP3α Block II and MSP9 N-terminus, further analyses examining the effect of antibody levels were restricted to these 2 proteins. Antibody positive children were stratified into 2 equal sized groups and designated as high or low responders. Children with high antibody levels to both PvMSP3α Block II (high: aIRR = 0.46, CI_95_[0.28, 0.77], low: aIRR = 0.72, CI_95_[0.45, 1.17]) and PvMSP9 N-Terminus (without concurrent infection, high: aIRR = 0.30, CI_95_[0.14, 0.62], low: IRR = 0.76, CI_95_[0.40, 1.45]), had a lower risk of clinical *P. vivax* malaria compared to children with low antibody levels but that difference was only significant for PvMSP9 N-Terminus (p = 0.04) and not PvMSP3α Block II (p = 0.15).

There were no significant associations observed with antibodies to any of the *P. vivax* proteins and risk of *P. falciparum* clinical episodes (p>0.16).

## Discussion

Antigens on the surface of the merozoite have long been considered as promising vaccine candidates based on their accessibility to the immune system. Antibodies against *P. vivax* merozoite surface proteins and their *P. falciparum* orthologues are thought to function by directly inhibiting invasion of erythrocytes, opsonising merozoites for uptake by phagocytes and through antibody-dependent cell-mediated immune mechanisms [19], [30], [40], [41], [42], [43], [44], [45]. Although studies have established the immunogenicity of a number of *P. vivax* merozoite antigens using serum from malaria exposed individuals [10], [12], [21], [27], [28], [31], [46], [47], [48], few have examined the contribution of antibodies to protective immune responses against *P. vivax* infection in malaria exposed populations [15], [27], [31], [49], [50]. Our results demonstrate that IgG specific responses against defined regions of PvMSP3α and PvMSP9 are significantly associated with protection from symptomatic *P. vivax* infection in young children resident in a malaria endemic region of PNG. Antibody responses against these proteins have been previously examined in malaria-exposed populations [27], [31] however, these studies were limited in their ability to precisely define the role of these antibodies. We employed a longitudinal study design with active screening for re-infection and morbidity and related antibody responses at baseline with prospective risk of developing symptomatic *P. vivax* infection over the 16-month follow-up period.

Overall, the prevalence of IgG specific responses to the different PvMSP3α and PvMSP9 antigens was low. Patterns of responsiveness to the recombinant proteins were different to that observed in previous studies [27], [31]. This is likely to reflect differences in age ranges, malaria transmission levels and potentially population genetics. However, we cannot rule out that differences in the reactivities of the non-exposed donors that were used to establish the threshold for seropositivity in the different studies may have contributed to disparity between studies. Although the age range of this cohort was narrow, the prevalence of antibodies against all PvMSP3α proteins and the PvMSP9 RI-RII domain increased with age (and presumably exposure). This was only significant however for antibodies against the N-terminal and the Block I and II regions of PvMSP3α. Interestingly, there was no apparent effect of age on the prevalence of antibodies against the PvMSP9 N-terminus. As this region of PvMSP9 is known to be highly conserved [29], [30] this may reflect the presence and recognition of conserved epitopes. Antibodies against all of the PvMSP3α and PvMSP9 antigens were also more commonly found in individuals with concurrent *P. vivax* infection, although this difference was only significant for Block I PvMSP3α and the PvMSP9 N-terminus. This effect of active parasite infection on antibody positivity reflects the induction and/or boosting of existing antibody responses.

After adjusting for age and exposure, associations with protection from symptomatic *P. vivax* malaria were seen for antibodies against the PvMSP3α Block II and the PvMSP9 N-terminal domains. This association remained following multivariate analyses. Additionally, antibodies against these proteins were associated with stronger reduction in risk of high compared to low infections. This density-dependent effect is in keeping with the proposed mechanism of action of these antibodies (i.e., to prevent/interfere with merozoite invasion of red blood cells and/or opsonisation of merozoites). This is most evident for PvMSP3α Block II, where higher levels of antibodies were associated with a significantly stronger protection compared with lower levels. Although PvMSP9 specific antibodies were more prevalent in children with concurrent infections, these antibodies were only associated with protection in children who did not have concurrent *P. vivax* infections at the time of antibody measurement. This indicates the existence of at least two separate types of antibodies targeting the PvMSP9 N-terminal domain: one that is easily boosted by infection and another that is longer lasting and continues to be present after an infection is cleared. Only the latter appear to be associated with protection against clinical *P. vivax* malaria. Whether these differential antibody types indicate the induction of different IgG subclass responses or target different epitopes is yet unknown.

A recent study demonstrated that the majority of sequences containing linear B cell epitopes within PvMSP3α are localised within the Block I and II repeat regions, although a few epitopes were detected in the more conserved N- and C-terminal flanking regions [27]. While the central domain of PvMSP3α is highly polymorphic, the C-terminal half containing the Block II repeat region is relatively conserved with only 2 major regions of polymorphism [26]. The PvMSP9 N-terminus is also known to be highly conserved across *Plasmodium* species [29], [30]. The conservation of these antigens amongst *P. vivax* isolates highlights their potential as vaccine candidates. Additionally, it has recently been shown that anti-sera generated against individual recombinant proteins representing the central alanine-rich domain of different PvMSP3 family members (including PvMSP3α), contain antibodies capable of recognising the central domain of other PvMSP3 family members [18]. While this suggests that an immune response generated against a single PvMSP3 protein may consist of a broad antibody response against multiple antigenic targets, further studies are required to determine whether the B cell epitopes recognised by these cross-reactive antibodies are contained within the C-terminal half of this domain. Studies to identify and characterise B cell epitopes that are targets of protective antibodies would inform the development and use of these molecules. Furthermore, investigating the functionality of antibodies against the PvMSP9 N-terminus and PvMSP3α Block II would confirm the utility and importance of these proteins as vaccine candidates. Undertaking *ex vivo* invasion assays with affinity purified serum containing antibodies specific for these proteins together with a range of *P.vivax* isolates would also shed light on the strain-transcending nature of the antibody response. In conclusion, these findings have significant implications for the development of a *P. vivax* specific vaccine. Controlling for _mol_FOB ensures that the observed associations of antibodies specific for the PvMSP9 N-terminus and PvMSP3α Block II with protection against *P. vivax* malaria in this study are not confounded by individual differences in exposure. Our results support further investigation of the PvMSP9 N-terminus and PvMSP3α Block II as vaccine candidates.
